# Redox-responsive PEGylated self-assembled prodrug-nanoparticles formed by single disulfide bond bridge periplocymarin-vitamin E conjugate for liver cancer chemotherapy

**DOI:** 10.1080/10717544.2017.1365393

**Published:** 2017-08-24

**Authors:** Huiyun Zhang, Wenqian Xu, Emmanuel Omari-Siaw, Yingkun Liu, Baoding Chen, Deyu Chen, Jiangnan Yu, Ximing Xu

**Affiliations:** a Department of Pharmaceutics, School of Pharmacy, Center for Nano Drug/Gene Delivery and Tissue Engineering, Jiangsu University, Zhenjiang, People’s Republic of China;; b Department of Ultrasound, The Affiliated Hospital of Jiangsu University, Zhenjiang, People’s Republic of China;; c Department of Radiation Oncology, The Affiliated Hospital of Jiangsu University, Zhenjiang, People’s Republic of China;; d School of Pharmacy, China Pharmaceutical University, Nanjing, People’s Republic of China

**Keywords:** Cardiac glycosides, periplocymarin, target delivery, cancer treatment, prodrug

## Abstract

Periplocymarin (PPM), a cardiac glycoside, has a narrow therapeutic index, poor tumor selectivity and severe cardiovascular toxicity which hinder its wide clinical applications in cancer treatment. Herein, we report novel redox-responsive prodrug-nanoparticles (MPSSV-NPs) self-assembled by co-nanoprecipitation of PPM-vitamin E conjugate and a PEG derivative of linoleate (mPEG2000-LA) in water. It was found that the characteristics of PPM-vitamin E nanoparticles (PSSV-NPs) were improved through co-nanoprecipitation with increased percentages of mPEG2000-LA. Moreover, the MPSSV-NPs were optimized according to the *in vitro* release and cytotoxicity study. Furthermore, the optimized MPSSV-NPs dramatically enhanced the circulation time and tumor distribution of PSSV-NPs after single intravenous injection. The *in vivo* studies in malignant H_22_-bearing mice revealed that MPSSV-NPs could effectively suppress tumor growth without causing obvious systemic toxicity. Altogether, these results suggested that MPSSV-NPs could offer a safe, multifunctional and viable nanoplatform for cardiac glycosides in cancer treatment.

## 1. Introduction

Cardiac glycosides are a kind of steroids that possesses strong cardiotonic effect. Chemically, cardiac glycosides are divided into two types namely cardenolides and bufadienolides. Recently, cardiac glycosides have been shown to exhibit great potential in inhibiting the growth of malignant tumor cells and inducing cancer cells apoptosis, and have attracted much attention in the prevention and treatment of cancer (Milutinovic et al., 2016; Diederich et al., 2017). Digoxinum, Strophanthin G, and UNBS1450 (semi-synthetic drug) have been subjected to clinical studies, but their therapeutic effect in cancer or tumor treatment are not satisfactory.

Periplocymarin (PPM), a cardiac glycoside isolated from *Cortex periplocae,* can inhibit proliferation of PC3, U937, HCT-8, Bel-7402, BGC823, A549, and A2780 cell lines *in vitro* with IC_50_ values of 0.02–0.29 µM (Bloise et al., 2009). It functions via inhibiting the activity of sodium (Na^+^)/potassium (K^+^)-ATPase (NKA) leading to cell death by apoptosis or necrosis (Newman et al., 2008). However, the clinical use of PPM in the treatment of cancer is challenged by many setbacks including cardiac toxicity, narrow therapeutic window, low drug delivery rate, and poor tumor-targeting (Zhang et al., 2016a). Overcoming these limitations could significantly advance the usefulness of PPM.

Carrier-based delivery of anticancer drugs has received much attention in recent years because of its potential in improving drug efficacy, reducing unwanted side effects and circumventing cellular accumulation mediated drug resistance (Cao et al., 2012; Zhu et al., 2014; Luo et al., 2015). Lately, pharmaceutical nanotechnology has been widely employed as a strategy to improve stability and reduce toxicity of cardiac glycosides. Li et al. reported bufadienolide-loaded oral submicron emulsion as a suitable delivery system for oral administration of bufadienolides, with satisfactory stability, superior antitumor efficacy and low toxicity (Li et al., 2015). Tian et al. also found that bufalin-loaded biotinylated chitosan nanoparticles (NPs) showed dramatically improved antitumor activity in MCF-7 human breast cancer xenograft model (Tian et al., 2014). However, the partially hydrophobic drug, PPM, which can be soluble in methylene chloride and aqueous solutions are hard to be formulated into some commonly used carriers via a simple method of solvent evaporation (Yi et al., 2012, 2013a). What is more, the disadvantages of carrier-based delivery systems such as low drug loading and release, carrier-induced toxicity and immunogenicity as well as complex synthesis manipulation need critical consideration (Yi et al., 2013b; Lu et al., 2015).

Drug self-delivery systems represent another promising strategy to realize intracellular delivery by themselves without the aid of additional nanocarriers (Gaudin et al., 2016; Luo et al., 2016). Among the self-delivery systems, nanoprodrug self-delivery systems have gradually attracted much attention due to its improved features in ensuring high and fixed drug content while preventing rapid clearance and premature burst release (Dastidar et al., 2016; Qin et al., 2016b). Based on the enhanced permeability and retention (EPR) effect, such delivery approaches often exploit the on-demand drug release in tumor attributed to the abnormal pH, redox potential, and overexpressed enzymes/specific secretions within cancerous tissues/cells (Liu et al., 2014; Cong et al., 2016; Li et al., 2016; Luo et al., 2016). Wang et al. developed a self-delivered and self-assembled nanomedicine through insertion of a disulfide bond between a water-soluble fluorescent probe, sulforhodamine B and vitamin E, which proved a promising theranostic tool for tumor imaging (Wang et al., 2014). Therefore, nanoprodrug self-delivery systems offer directions for water-soluble cardiac glycosides nanoscale tumor-target, like PPM, proscillaridin, and scillaren.

In this study, we rationally designed novel redox-responsive prodrug-nanoparticles (MPSSV-NPs) self-assembled by co-nanoprecipitation of PPM-S-S-vitamin E conjugate (PPM-S-S-VE) and a PEG derivative of linoleate (mPEG2000-LA) in water. Importantly, we found that the amphiphilic PEG derivatives could improve the stability and redox-responsiveness of NPs by creating a PEG brush on the NP surfaces. Furthermore, the *in vitro* and *in vivo* anticancer activities of the optimized PEGylated prodrug-nanoparticles (MPSSV-NPs) were evaluated comparatively to the non-PEGylated PPM-vitamin E nanoparticles (PSSV-NPs).

## 2. Materials and methods

### 2.1. Materials

Bufalin, mPEG2000, and linoleic acid were obtained from J&K Scientific Co., Ltd. (Beijing, China). Dicyclohexylcarbodiimide (DCC), 4-dimethylaminopyridine (DMAP), acetic anhydride, vitamin E, and dithiodiglycolic acid were obtained from Aladdin Industrial Corporation (Shanghai, China). 3-(4,5-Dimethylthiazol-2-yl)-2,5-diphenyltetrazolium bromide (MTT) and trypsin were purchased from Beyotime Institute of Biotechnology (Jiangsu, China). Fetal bovine serum and Dulbecco’s modified Eagle’s medium (DMEM) were purchased from Gibco Company (Grand Island, NY). Chromatographically pure methanol and acetonitrile were obtained from Hanbon Technology Co., Ltd. (Jiangsu, China).

### 2.2. Synthesis of PPM-S-S-VE

Dithiodiglycolic acid (0.2 g, 1.10 mmol) was dissolved in 3 mL anhydrous acetic anhydride and stirred for 2 h at 30 °C. Excess solvent was then evaporated with addition of toluene to dryness under high vacuum. The residue was dissolved in dichloromethane (2 mL), and then VE (0.1 g, 0.23 mmol) and a catalytic amount of DMAP was added. The reaction was stirred at room temperature for 2 h. The product (vitamin E-dithiodiglycolic acid, VE-S-S-COOH) was purified by silica gel column chromatography, eluting with a solution of hexane, ethyl acetate, and acetic acid (V/V/V = 10:1:1) to obtain a yellow oil (85 mg, 61.6%).

VE-S-S-COOH (120 mg, 0.20 mmol), DCC (48 mg, 0.23 mmol) and DMAP (30 mg, 025 mmol) and PPM (100 mg, 0.19 mmol) were added to dichloromethane (6 mL). The solution was stirred for 2 h, filtered to remove DCU and purified by silica gel column chromatography. The elution solution was petroleum ether alongside ethyl acetate (V/V = 2:3) and a white solid (80 mg, 37.6%) target compound (PPM-S-S-VE) was obtained.

### 2.3. Synthesis of mPEG2000-LA

Linoleic acid (0.5 g, 1.78 mmol), mPEG2000 (1 g, 0.5 mmol), DCC (0.4 g, 1.94 mmol), and DMAP (0.24 g, 1.96 mmol) were added to dichloromethane (6 mL) at room temperature. The solution was stirred for 2 h under nitrogen atmosphere, filtered to remove DCU, washed with saturated sodium chloride solution, dried over Na_2_SO_4_ and concentrated *in vacuo*. The residue was washed with petroleum ether thrice and dried under vacuum to give mPEG2000-linoleic acid (mPEG2000-LA) as a white solid (1.09 g, 62%).

### 2.4. Preparation of prodrug nanoparticles

NPs were prepared as previously described by nano-precipitation (Gaudin et al., 2015). Briefly, the tested compound was dissolved in ethanol and then added drop-wise into water under mechanical stirring (∼600–800 rpm) at room temperature, to give a final ethanol concentration of 2–5% ethanol. Under these conditions, the self-assembly of PSSV-VENPs occurred spontaneously. Ethanol in the nano-formulation was then evaporated under vacuum at room temperature, and the organic solvent-free prodrug NPs were stored at 4 °C. PEGylated PPM-S-S-VE NPs were produced by co-nanoprecipitation of PPM-S-S-VE and mPEG2000-LA, with different weight ratio (mPEG2000-LA/PPM-S-S-VE = 1/10, 1/5, 2/5, 4/5, 5/5, w/w) added to the ethanolic phase, before drop-wise addition to the aqueous phase.

### 2.5. Stability test

The long-term stability of prodrug NPs was investigated by measuring the mean diameter of the NPs. Briefly, prodrug NPs with a final concentration of 0.5 mg/mL were incubated in water and PBS solution (pH = 7.4) at 25 °C for a month. Particle size was measured at pre-determined time intervals (1, 2, 3, 5, 10, 15, 20, 25, and 30 d).

### 2.6. Characterization of prodrug nanoparticles

The particle size distribution and zeta potential of prodrug NPs were measured using dynamic light scattering (DLS) method via Zeta Potential/Particle Size analyzer (Brookhaven Instruments Corporation, Holtsville, NY). The morphological properties of the NPs were examined using transmission electron microscope (TEM) (JEM-2100, JEOL, Tokyo, Japan) at an acceleration voltage of 200 kV. A drop of diluted NPs solution (0.5 mg/mL) was put on a copper grid and air-dried at room temperature. The sample was stained with phosphotungstic acid solution (2%, w/v) and subsequently viewed under the TEM.

### 2.7. *In vitro* drug release and degradation profile

*In vitro* release of PPM from prodrug NPs and the degradation of PPM-S-S-VE conjugate were performed under Water-bathing Constant Temperature Vibrator (SHZ-88, Jintan Medical Instrument Corporation, Zhejiang, China) with shaking (100 rpm) at 37 °C in four different media namely phosphate buffer saline (PBS, pH 7.4) containing 10 mM, 1 mM, 10 μM, and 1 μM GSH, respectively. An aliquot (0.1 mL, 1 mg•mL^−1^) of MPSSV-NPs was transferred to 2.5 mL of the release media. At certain time intervals (0, 0.5, 1, 2, 4, 6, 8, 12, and 24 h), 0.1 mL release media were taken and subsequently replaced with equal volume of fresh media (preheated to 37 °C). The collected samples at each time point was added to 0.3 mL methanol for high-performance liquid chromatography (HPLC) analysis. The content of PPM and PPM-S-S-VE conjugate were determined by HPLC with Shimadzu Scientific instrument equipped with an LC-20AT pump and an SPD-20A UV-Vis detector (Shimadzu, Kyoto, Japan) on a reverse ODS Symmetric-C18 column (150 mm × 4.6 mm, 5 μm) with thermostat at 30 °C and UV detection at 220 nm using a mixture of acetonitrile/water as a mobile phase as well as a flow rate of 1 mL/min. Data were presented as means of triplicate samples.

### 2.8. *In vitro* cytotoxicity assay

Different cancer cell lines (HepG2 and MCF-7 cells) in logarithmic growth phase were seeded at 4000 cells/well in DMEM supplemented with 10% (v/v) fetal calf serum, 100 U/mL penicillin, and 100 µg/mL streptomycin in 96-well plates. The preparation was cultured at 37 °C with 5% CO_2_ under fully humidified conditions. Following 24-h incubation, the cells were treated with 100 µL medium containing each of the respective drug samples (free PPM, PSSV-NPs, and MPSSV-NPs), with each having different concentrations (0.005–15 µM). After 72-h incubation, MTT solution (20 µL, 5 mg/mL) was added to each well, and the plates incubated further at 37 °C for 4 h. DMSO (100 µL) was used to solubilize the formazan crystals and the optical density measured on microplate reader at 595 nm. The cell viability rate (VR) was calculated according to the equation:

$$\text{VR }\left(\%\right)\;=\;A/A^{1}\times 100\%$$

where *A* refers to the absorbance of treated group and *A*^1^ refers to the absorbance of untreated control group (the vehicle control group).

### 2.9. Pharmacokinetic study

The animal experiments were conducted according to the guidelines of the Jiangsu Council on Animal Care (approval number: 2013-0036). Fifteen male SD rats (220 ± 20 g), randomly and equally divided into three groups, were injected via lateral saphenous vein with PPM solution, PSSV-NPs and MPSSV-NPs of the equivalent PPM dose (4 mg/kg, *n* = 5), respectively. Blood samples (500 µL) were collected at predetermined time points (0.25, 0.5, 1, 1.5, 2,4, 6, 8, 12, and 24 h), and centrifuged to obtain the serum. Serum samples (200 µL each) were taken and added to 50 µL of 1 μg·mL^−1^ bufalin (internal standard). Then, ethyl acetate (1.2 mL) was added to the mixture, vortex mixed for 3 min and centrifuged at 3000 rpm for 10 min. The organic upper layer was removed and dried with nitrogen at 40 °C on a water bath to obtain the residue, which was later reconstituted in 100 µL methanol and vortex-mixed for 3 min. Again, the sample was centrifuged at 20,000 rpm for 10 min and the supernatant was used in HPLC analysis. The mobile phase consisted of acetonitrile and water (34:66, v/v, containing 0.05% TFA). The flow rate was 1.0 mL·min^−1^ and the detection wavelength was 220 nm. Other conditions included injection volume (20 µL), column temperature (25 °C) and detection sensitivity (0.02 AUFS). The retention time of PPM and bufalin was about 9.3 and 14.6 min, respectively. The calibration curve was *A* = 0.00086 *C* + 0.01006 (*A* represented the peak area ratio of PPM and bufalin, while *C* was the concentration of PPM) and was linear in the range of 54.24–5050 ng·mL^−1^ with a correlation coefficient of *R* = 0.9991.

### 2.10. Tissue distribution in H_22_ cell tumor bearing mice

The tissue distribution of prodrug NPs was assessed using tumor-bearing H_22_ mice (Kunming mice, weight 18–20 g, Laboratory Animals Care and Use of Jiangsu University, Jiangsu, Zhenjiang, China). A tumor xenograft model was prepared by subcutaneously injecting 1 × 10^7^ H_22_ hepatocarcinoma cells (100 µL) into the right hind leg. When the tumor volume reached 1000 mm^3^ (around seven days), mice were intravenously injected with PPM solution, PSSV-NPs and MPSSV-NPs of the equivalent PPM dose (4 mg/kg, *n* = 5), respectively. At indicated time periods (1, 2, and 4 h) after injection, blood samples were collected and mice were then sacrificed by cervical dislocation to obtain the heart, liver, spleen, lung, kidney, and tumor tissues. All the samples were then washed with ice-cold saline to remove the excess fluid, weighed and stored at −20 °C. The thawed tissue was homogenized in 0.9% sodium chloride solution to obtain 0.2 g/mL tissues homogenate. This was followed by the addition of bufalin (50 µL, 5 µg/mL) to the tissues (0.2 mL) with uniform mixing. Ethyl acetate (1.2 mL) was then added to the resulting mixture and thoroughly mixed for 5 min. The total supernatant or organic layer was separated by centrifugation at 3000 rpm for 10 min and transferred into a clean tube. The supernatant was dried with nitrogen at 40 °C on a water bath to obtain the residue, which was later reconstituted in a 0.2 mL of mobile phase solution. After centrifugation at 20,000 rpm for 10 min, the supernatant was analyzed by HPLC as described in a previous report (Zhang et al., 2016b).

### 2.11. Anti-tumor efficacy and toxicity

A tumor xenograft model was prepared as described in section 2.10. The administration to mice started when the tumor diameter reached about 0.5 cm at day 3 followed by treatment at every other day for five times. Mice were randomly divided into four groups (*n* = 8–9) namely the control, PPM, PSSV-NPs, and MPSSV-NPs groups. Treatment was administered via tail vein at a dose of 4 mg drug/kg. The control group received corresponding amounts of PBS (pH =7.4) solution. Their weights were measured daily and tumor size was measured (major and minor axis) with a vernier caliper thereafter and at the end of the experiment. Tumor volumes were calculated using the formula: *a*^2^×*b* × 0.52, where a and b refers to the longest and shortest diameter, respectively. Tumor tissues were removed from the sacrificed mice and weighed to calculate the tumor inhibition rate (TIR (%) = (1 – Wt/Wc) × 100%), where Wt and Wc are the mean tumor weights of the treated groups and the negative control group, respectively. Moreover, the heart, liver, and tumor were rinsed with normal saline, fixed in 10% formalin, and then embedded in paraffin blocks for later slicing and staining with hematoxylin and eosin (H&E).

## 3. Results

### 3.1. Synthesis of PPM-S-S-VE and mPEG-LA

Periplocymarin was conjugated with vitamin E via disulfide bond as described in previous studies (Scheme S1) (Xue et al., 2016). The chemical identities of the two conjugate were confirmed by ^1^H NMR (Figures S1–3), HPLC and ESI-MS (Figure S4, 5). The first step was to convert dithiodiglycolic acid to the corresponding anhydride with acetic anhydride.

Next, it was reacted with vitamin E to generate VE-S-S-COOH. Chemical formula C_33_H_4_O_5_S_2_, ESI-MS (*m*/*z*): 595.43 [M + H]^+^, 612.42 [M + NH_4_]^+^, 617.63 [M + Na]^+^. ^1^H NMR (400 MHz, CDCl_3_, ppm): *δ* 3.90 (2H, s), 3.70 (2H, s), 2.63 (2H, m), 2.13 (3H, s), 2.08 (3H, s), 2.04 (3H, s), 1.89–1.75 (2H, m), 0.85–0.94 (12H, m). Finally, VE-S-S-COOH was coupled to PPM to yield PPM-S-S-VE, with the assistance of DCC and DMAP. Chemical formula C_63_H_98_O_12_S_2_. ESI-MS (*m*/*z*): 1134.14 [M + Na]^+^. ^1^H NMR (400 MHz, CDCl_3_, ppm): *δ* 5.90 (1H, s, H-22), 5.00 (1H, dd, *J* = 1.6, 18.4 Hz, H-21 b), 4.89 (1H, dd, *J* = 2.0, 9.6 Hz, H-1′), 4.83 (1H, dd, *J* = 1.6, 18.4 Hz, H-21a), 4.17 (1H, m, H-3), 3.88 (2H, s, –SCH_2_CO_2_–PPM), 3.70 (2H, s, –SCH_2_CO_2_–VE), 3.44 (1H, m), 3.40 (3H, s, CH_3_-7′), 2.63 (2H, m), 0.85–0.94 (24H, m).

mPEG2000-LA was simply synthesized through the direct esterifying reaction of mPEG2000 and linoleic acid (Scheme S2). The yield of mPEG2000-LA was 62% and the results of spectroscopic analyses were as follows: ^1^H NMR (400 MHz, CDCl_3_, ppm): *δ* 5.23 (6H, m), 4.15 (2H, t), 3.58 (180H, s), 3.31 (3H, s), 3.11 (2H, s), 2.69–2.72 (2H, t), 2.25–2.32 (2H, t), 1.84–1.96 (4H, s), 1.18–1.38 (15H, m), 0.83 (3H, t). As Figure S6 shows, the highest *m*/*z* of mPEG2000 was 1005.5, which represents 1/2 *M* + 1. It was speculated that the number average molecular weights (*Mn*) of mPEG2000 was 2008, and after conjugation with LA, the number average molecular weights (*Mn*) of mPEG2000-LA was 2226, which was speculated from the highest *m*/*z* 1114.59 (Figure S7). The results showed that number average molecular weights of mPEG2000-LA increased by 218 as compared to that of mPEG2000. However, the determined *Mn* values of mPEG2000-LA was 44 lower than the theoretical values (*Mn* = 2270). Such a gap of 44 was exactly the *Mn* of single PEG unit (C2H4O), which could result from the loss of higher *Mn* fractions of mPEG2000-LA during purification. Therefore, the triple TOF-MS results could confirm the successful synthesis of mPEG2000-LA.

### 3.2. Preparation and characterization of PSSV-NPs and MPSSV-NPs

**Figure 1.**
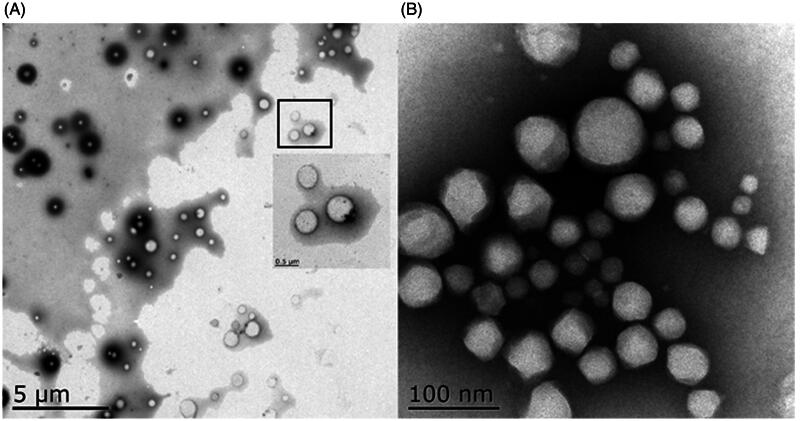
TEM of PSSV-NPs (A) and MPSSV-NPs (B).

PSSV-NPs was prepared by nano-precipitation of an ethanolic solution of PPM-S-S-VE to distilled water. PPM-S-S-VE NPs could ensure a high drug loading (48%) according to the molecular weight ratio of the drug versus the prodrug (Ralayranaivo et al., 2014). Different amounts of mPEG2000-LA were incorporated with PPM-S-S-VE to self-assemble to form PEGylated PPM-S-S-VE NPs, while keeping the final concentration of PPM-S-S-VE constant. The stability of prodrug NPs was evaluated in water and PBS at 25 °C. It showed that both PSSV-NPs and MPSSV-NPs were remarkably stable upon incubation in water for a five-day period (Figure S8a). But over time, PSSV-NPs and MPSSV-NPs at lower PEG contents aggregated slowly. This phenomenon of aggregation was also highlighted with PBS (Figure S8b). Fortunately, MPSSV-NPs with ratios up 1:0.2 remained stable in water and PBS solution for one month. DLS analysis revealed that the PSSV-NPs had a size of 230.54 ± 10.23 nm, PDI of 0.085 ± 0.008, and zeta potential of –28.42 ± 1.23 mV (Table S1 and Figure S9). However, the diameter and zeta potential of the particles decreased with the addition of mPEG-LA in the formulation till 1:0.8, and was 137 nm and –23.16 mV, respectively. The TEM micrograph (Figure 1) showed that PSSV-NPs and MPSSV-NPs (1:0.8) were spherical and the particle sizes were nearly 230 nm and 110 nm, respectively, which corroborated with the results detected by DLS. What is more, the PEG corona was found on the surface of MPSSV-NPs (1:0.8).

### 3.3. *In vitro* release study

**Figure 2.**
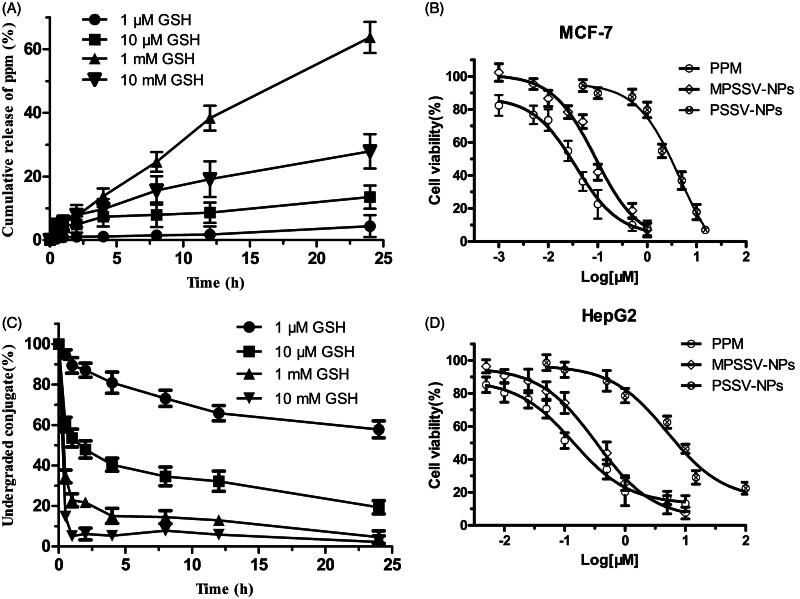
*In vitro* release studies. The GSH-sensitive release of PPM (A) and degradation of PPM-S-S-VE conjugate (C) from MPSSV-NP were studied at 37 °C under four different conditions, i.e. 10 mM, 1 mM, 10 μM, and 1 μM GSH, respectively (*n* = 3); *in vitro* cytotoxicity of PSSV-NPs and MPSSV-NPs with various concentration on MCF-7 (B) and HepG2 (D) cells after 72 h of incubation. (error bars are mean ± SD, *n* = 3).

*In vitro* redox-responsivity of PSSV-NPs and MPSSV-NPs was investigated at 37 °C in PBS (pH =7.4) with four GSH conditions. The results showed that the release of PPM from NPs in the presence of 1 mM GSH increased with additional amount of mPEG-LA in the formulation till 1:0.8 (Figure S10). More than 50% of the total amount of PPM was released from MPSSV-NPs (1:0.8) in 24 h. In contrast, only 25.63% of PPM was released from PSSV-NPs under the same condition. Therefore, the sensitivity to GSH increased as the amount of mPEG-LA increased on the surface of NPs until completely covered. In addition, the PPM release and PPM-S-S-VE degradation of MPSSV-NPs (1:0.8) exhibited redox-responsive drug release in the presence of GSH stimuli (Figure 2(A,C)). What is more, the drug release in 1 mM GSH is faster than that of in 10 mM GSH due to the instability of PPM in 10 mM GSH (Figure S11).

### 3.4*. In vitro* cytotoxicity assay

We further compared the *in vitro* cytotoxicity of PSSV-NPs and MPSSV-NPs on MCF-7 and HepG2 cells by the MTT assay using PPM as controls. The cell viability of different kinds of cells are shown in Figure 2(B,D). The IC_50_ values of different nanoprodrug against the two cell lines are calculated and summarized in Table S2. Although, the cytotoxicity of PSSV-NPs and MPSSV-NPs in MCF-7 (IC_50_ were 4.517 and 0.0897 μM, respectively) were lower than that of PPM (IC_50_ was 0.03594). After co-precipitating with mPEG-LA, the cytotoxicity of PSSV-NPs was significantly improved and even close to free PPM. We also discover same phenomenon on HepG2 cells.

### 3.5. Pharmacokinetics of PSSV-NPs and MPSSV-NPs

The *in vivo* pharmacokinetic results demonstrated that PSSV-NPs and MPSSV-NPs had a lower maximum plasma concentration (*C*_max_) than PPM and this could provide greater tolerance dose due to the very narrow therapeutic window of PPM (Figure S12). Moreover, PSSV-NPs and MPSSV-NPs had a prolonged circulation time with an elimination phase half-life (*t*_1/2_) of 3.339 and 30.04 h, respectively, compared to PPM with 0.327 h (as shown in Table S3). MPSSV-NPs exhibited an obviously longer circulation time than PSSV-NPs, which is likely due to the mPEG-LA on the surface of MPSSV-NPs and the instability of PSSV-NPs. At the same time, it indicated that mPEG-LA was stable to keep the structural stability of MPSSV-NPs in blood. Thus, MPSSV-NPs had a significantly higher area under the blood concentration curve (AUC) of 46.8 μg/mL·h, which were 21 and 9 times higher than those of the free PPM and PSSV-NPs, respectively.

### 3.6. Biodistribution in H_22_ liver tumor-bearing mice

**Figure 3.**
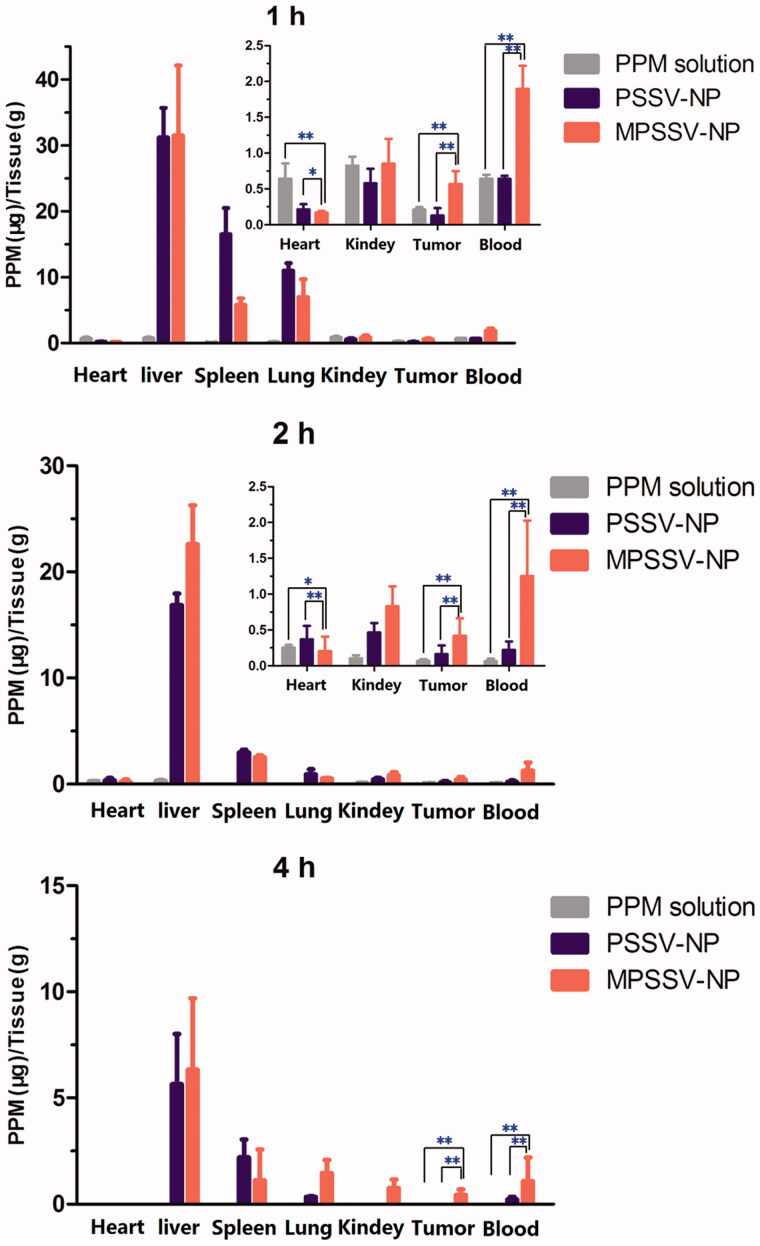
Tissue distribution of PPM after intravenous administration of PSSV-NPs and MPSSV-NPs injection in H_22_-bearing mice in blood, tumor, liver, spleen, lung, kidney, and heart (*p < .05, **p < .01, and n − 5 for each time point).

In this study, the *in vivo* biodistribution of PSSV-NPs and MPSSV-NPs following intravenous administration in H_22_ tumor bearing mice was investigated. Mice were sacrificed at 1, 2, and 4 h, post injection and tissues were harvested to determine the level of PPM. The results of PPM concentration in tissues at predetermined time intervals for each group are exhibited in Figure 3. At 1 h post injection, the PPM concentration at the liver, spleen, and lung in MPSSV-NPs and PSSV-NPs groups were higher than those of PPM group due to the higher blood concentration and the capture from reticulo endothelial system (RES) (Soenen et al., 2013; Lin et al., 2016). Due to the EPR effect, the accumulations of MPSSV-NPs and PSSV-NPs at tumor were obviously higher than PPM. However, accumulation in the heart decreased. Compared to PSSV-NPs, less accumulation of MPSSV-NPs in spleen and lung was found while the liver and tumor drug concentrations were higher owing to the longer circulation time as shown in the *in vivo* pharmacokinetic study. At 4 h post injection, PPM in the free PPM group could barely be detected in the blood and tumor. In the case of the MPSSV-NPs group as compared to the PSSV-NPs group, although there was still a high level of drug concentration in the liver, the free drug was detected at the tumor site at desired time intervals. This matches with other reports which indicated that free PPM is mainly distributed in the liver and heart (Yan et al., 2016). As for the MPSSV-NPs group, the drug concentration in the liver was gradually reduced and barely detectable in the heart.

### 3.7. *In vivo* anti-tumor efficacy and toxicity of MPSSV-NPs

**Figure 4.**
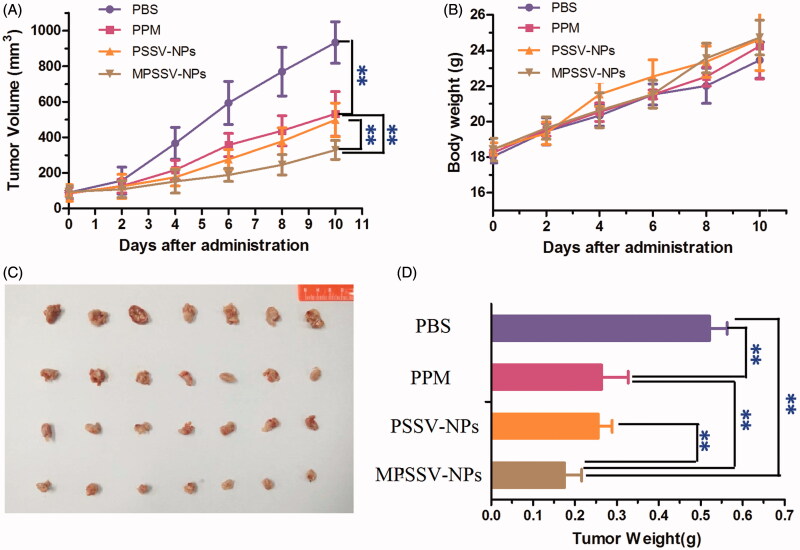
Graph showing tumor volume of H_22_ tumor-bearing mice in different treatment groups. (A) Antitumor effect in terms of tumor growth; (B) the change of body weight during the treatments; (C) tumor growth after systemic application of different treatment groups; (D) tumor weight (**p < .01, and n − 8 for each time point).

**Figure 5.**
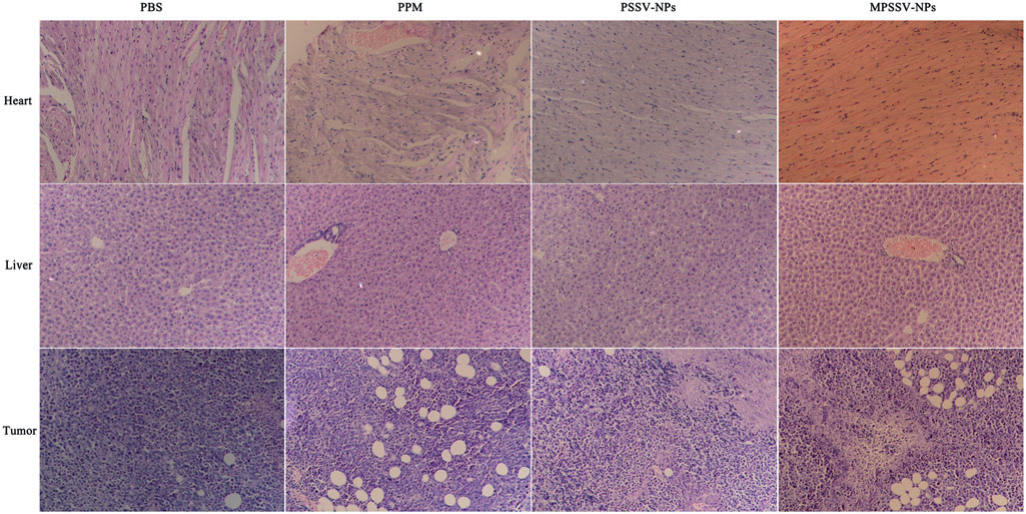
Typical histopathologic images of rat hearts, livers, and tumors after treatment in H_22_-bearing mice with PSSV-NPs and MPSSV-NPs group for 10 days (H&E staining, ×20).

From the above results, MPSSV-NPs showed more stability, redox-sensitivity, *in vitro* anti-cancer activity, and tumor targeting. The *in vivo* antitumor activity was further evaluated in Kunming mice bearing H_22_ tumor. As presented in Figure 4, it was demonstrated that MPSSV-NPs was more efficient in suppressing growth of tumors compared with free PPM and PSSV-NPs. According to Figure 4(D), tumor weight in MPSSV-NPs group (0.175 ± 0.041 g) was significantly lower than that of the free PPM (0.263 ± 0.064, *p* < .01), PSSV-NPs (0.256 ± 0.033 g, *p* < .01), or control group (0.521 ± 0.042, *p* < .01). Furthermore, according to the histopathological examination of the tumors under a microscope (Figure 5(C)), MPSSV-NPs exhibited larger degeneration, disruption and death area than the free PPM and PSSV-NPs, whereas the control group showed closely spaced tumor cells with hyperchromatic nuclei. Importantly, MPSSV-NPs could reduce the heart toxic effects of PPM, which was the major toxicity, caused by cardiac glycosides (as shown in Figure 5(A)). Although, MPSSV-NPs were easy to accumulate in the liver, no obvious damages and pathological changes were found in the liver sections compared with the control group (Figure 5(B)).

## 4. Discussion

In our study, the PPM-vitamin E conjugate was successfully synthesized, and could self-assemble into prodrug nanoparticles (PSSV-NPs) through the insertion of a disulfide bond to balance intermolecular forces. However, in agreement with already reported data, intermolecular forces in this system were still instable in PBS solution (Langenhan et al., 2005; Wang et al., 2014). Therefore, we designed a PEG derivative of linoleate (mPEG2000-LA) to co-precipitate with PPM-vitamin E conjugate in order to balance intermolecular forces and obtain a PEGylated self-assembled prodrug-nanoparticles (MPSSV-NPs). Characterization of MPSSV-NPs including particle size, zeta potential, morphology, stability was measured. Several reports have indicated that PEGylated prodrug NPs have bigger size than non-PEGylated prodrug NPs due to the surface of prodrug NPs covered by DSPE-PEG2000 or TPGS2000 (Cong et al., 2016; Xue et al., 2016). However, our results indicated that MPSSV-NPs have smaller size, higher zeta potential and more stability than PSSV-NPs. The reasons for smaller particle size could be that mPEG2000-LA was efficiently incorporated in prodrug NPs and could reduce surface tension between the aqueous phase and the organic phase (Wang et al., 2015). In addition, PEG formed a coating ‘brush’ on the surface of NPs could prevent particle aggregation by collision and hydrophobicity (Bekkara-Aounallah et al., 2008).

Potential targeted drug delivery systems should be able to overcome not only extracellular barriers (long circulation time, preferential accumulation at diseased sites, selective binding to the targeted cells, etc.) but also equally important intracellular barriers (cellular internalization, endosomal escape, drug release, etc.) (Xu et al., 2015). *In vitro* studies showed that MPSSV-NPs (1:0.8) exhibited better redox-responsive drug release in the presence of GSH stimuli than PSSV-NPs. This could be attributed to the hydrophobic properties of PPM-S-S-VE making that the disulfide bond of PPM-S-S-VE unsusceptible to attack by GSH (Wang et al., 2015). However, increasing percentage of mPEG-LA correlated enhanced stability and hydrophilicity of PSSV-NPs. Therefore, MPSSV-NPs would have stronger targetability and cytotoxicity on tumor cells as it contain a higher concentration of glutathione (GSH, 2–8 mM) than normal cells (Qin et al., 2016a). *In vitro* cytotoxicity results indicated that MPSSV-NPs was more effective in killing cancer cells due to an improved intracellular penetration, stability and redox-responsiveness (Langenhan et al., 2005; Otsuka et al., 2012).

The *in vivo* pharmacokinetic results indicated that MPSSV-NPs exhibited a good pharmacokinetic profile compared with PPM and PSSV-NPs. Narrow therapeutic window and rapid metabolism would hinder the development of PPM in clinical application (Haux, 1999). A lower maximum plasma concentration (*C*_max_) makes MPSSV-NPs safer than PPM. Furthermore, as the residence time of MPSSV-NPs in the body extended, the tumor accumulation was improved due to the longer circulation than PSSV-NPs. Furthermore, the biodistribution results in H_22_ liver tumor-bearing mice indicated that MPSSV-NPs could enhance the drug retention time and increase concentration in tumor tissue. On the other hand, MPSSV-NPs could decrease the accumulation of PPM in heart. It means that MPSSV-NPs could decrease heart toxicity of PPM. Pathological images of hearts proved that MPSSV-NPs have lower heart toxicity than PPM. Taken together, these results consistent with the *in vitro* stability and pharmacokinetic studies described above, showed that the enhanced stability and PEGylated surface conferred by mPEG-LA avoided the phagocytosis of NPs by immune system, and consequently contributed to higher intratumoral accumulation (Gong et al., 2010; Sha et al., 2015). The results of *in vivo* anti-tumor efficacy and toxicity finally proved that MPSSV-NPs, benefiting from PEGylated longer circulation in body, accumulation in tumor through EPR and redox-responsive release in tumor microenvironment, showed markedly improved therapeutic effects compared to free PPM and PSSV-NPs (Ramasamy et al., 2014).

Recently, PPM and other cardiac glycosides in nontoxic concentrations have been shown to inhibit proliferation and induce apoptosis and autophagy in different malignant cell lines *in vitro* (Li et al., 2012; Denicolaï et al., 2014). Cardiac glycosides are looking forward to become a new generation of anti-cancer drugs. To date, three cardiac glycosides products including Anvirzel™, PBI-02504, and UNBS1450 have been developed for treatment of cancer and were assessed in a phase I clinical trial (Mijatovic et al., 2007). However, currently, there were still many difficulties and problems in the practice of preclinical and clinical settings such as narrow therapeutic window, low drug delivery rate and poor tumor-targeting (Prassas & Diamandis, 2008). The solution to these problems mainly includes structure modification, nanotechnology, and use of prodrug. In our study, MPSSV-NPs fully exert and discover the advantages of above three methods. After structure modification with vitamin E to PPM-vitamin E conjugate, the toxicity of PPM would be obviously decreased according to the structure–activity relationship of cardiac glycoside and our *in vitro* study (Figure 4) (Moreno et al., 2013). However, as a prodrug-nanoparticles, MPSSV-NPs could be effectively delivered to tumor for on-demand release of PPM.

## 5. Conclusions

In summary, we have developed novel prodrug-nanoparticles (MPSSV-NPs) self-assembled by co-nanoprecipitation of PPM-S-S-vitamin E conjugate and a PEG derivative of linoleate (mPEG2000-LA) in water. The optimized MPSSV-NPs was more stable, redox-responsive, and low cytotoxic. What is more, it dramatically enhanced the circulation time and tumor distribution of PPM-vitamin E nanoparticles (PSSV-NPs). The *in vivo* studies in malignant H_22_-bearing mice proved that MPSSV-NPs could effectively suppress tumor growth without causing obvious systemic toxicity. The results suggested that MPSSV-NPs could offer a safe, multifunctional and viable nanoplatform for cardiac glycosides in cancer treatment.

## Supplementary Material

IDRD_Xu_et_al_Supplemental_Content.doc
