# Supplementary material for: Redox-responsive PEGylated self-assembled prodrug-nanoparticles formed by single disulfide bond bridge periplocymarin-vitamin E conjugate for liver cancer chemotherapy
Source: Drug Deliv. 2017 Aug 24;24(1):1170–8. doi: 10.1080/10717544.2017.1365393 (PMC8241199; doi:10.1080/10717544.2017.1365393)
Supplement: IDRD_Xu_et_al_Supplemental_Content.doc [file IDRD_A_1365393_SM2340.doc]

**Supplement**

**Redox-responsive PEGylated self-assembled prodrug-nanoparticles formed by single disulfide bond bridge** **periplocymarin-vitamin E conjugate for liver cancer chemotherapy**

Huiyun Zhang^a^, Wenqian Xu^a^, Emmanuel Omari-Siaw^a^, Yingkun Liu^a^, Baoding Chen^b^, Deyu Chen^c^, Jiangnan Yu^a,d^ and Ximing Xu^a^,*

^a^Department of Pharmaceutics, School of Pharmacy, Center for Nano Drug/Gene Delivery and Tissue Engineering, Jiangsu University, Zhenjiang 212013, People’s Republic of China; ^b^Department of Ultrasound, ^c^Department of Radiation Oncology, the Affiliated Hospital of Jiangsu university, Zhenjiang 212013, People’s Republic of China, ^d^School of Pharmacy, China Pharmaceutical University, Nanjing, People’s Republic of China

***Corresponding Author’s:**

Ximing Xu^a*^, 301 Xuefu Rd, Zhenjiang, Jiangsu 212013, P.R. China. Phone: 86-511-85038451; Fax: 86-511-85038451; E-mail: [xmxu@ujs.edu.cn](mailto:xmxu@ujs.edu.cn)

Jiangnan Yu^a,b*^, 301 Xuefu Rd, Zhenjiang, Jiangsu 212013, P.R. China. Phone: 86-18 511-8503845; Fax: 86-511-8503845; E-mail: yjn@ujs.edu.cn

**Methods**

1. ESI-MS

The ESI-MS data was acquired in a positive ion mode using a Thermo Finnigan LXQ Dec XP and Ion Trap Mass Spectrometer instrument from Thermo Finningan (San Jose, CA). The instrument was equipped with an electrospray ion source (ESI) and an Xcalibur® system manager data acquisition software. Sample solutions (2 µL, 5 mg/mL) were injected into the ESI source using a syringe pump with a mass scan range of 100-1000 m/z. The conditions for the ion trap mass spectrometer were as follows: spray voltage, 4.5 kV; source current, 80 µA; capillary temperature, 325 ^o^C; capillary voltage, 30 V; tube lens offset, 120 V; multi-pole 1 offset, 6 V; multi-pole 2 offset, 10 V; and sheath gas flow (N_2_), 35 A.U.

2. Chromatographic conditions

A Shimadzu instrument equipped with Inertsil ODS-SP C18 column (5 μm, 4.6×150 mm), an LC-20AT pump coupled to column compartment and SPD-20A UV/Vis Detector (Shimadzu, Kyoto, Japan) was used for the HPLC analysis. The LC solution workstation software (Shimadzu, Japan) was employed for data acquisition and analysis. The mobile phase for VE-S-S-COOH was consisted of 0.05% trifluoroacetic acid in methanol and water (95:5, v/v) with the flow rate of 1.0 mL/min. The monitored wavelength was 284 nm.

**The file includes**

1. Figure S1-11
2. Table S1


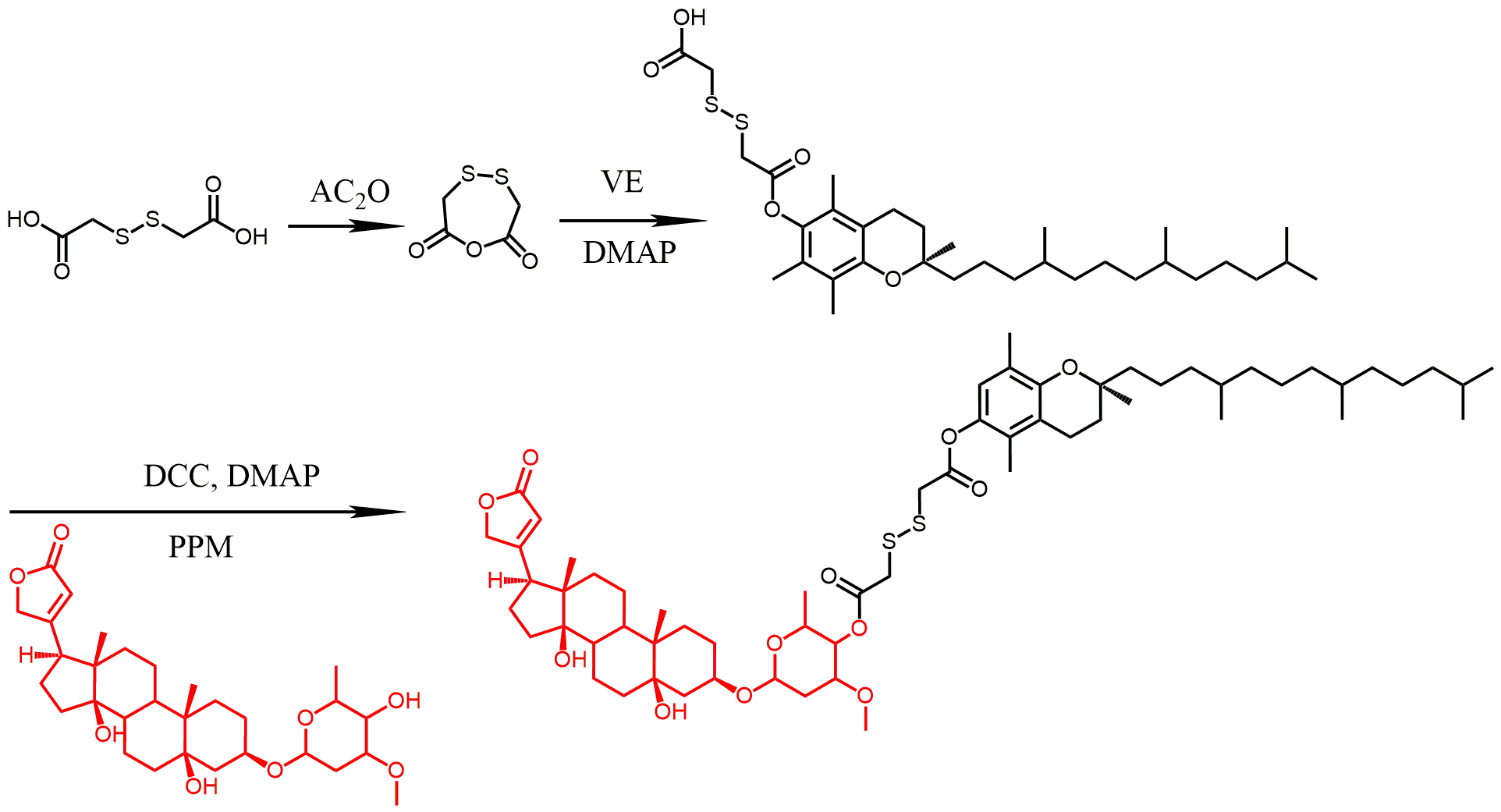


Scheme S1. Synthesis route of PPM-S-S-VE.


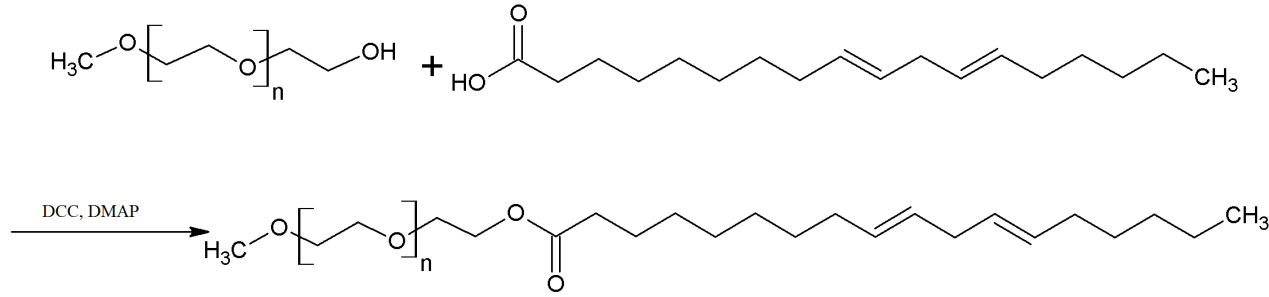


Scheme S2. Synthesis route of mPEG_2000_-LA


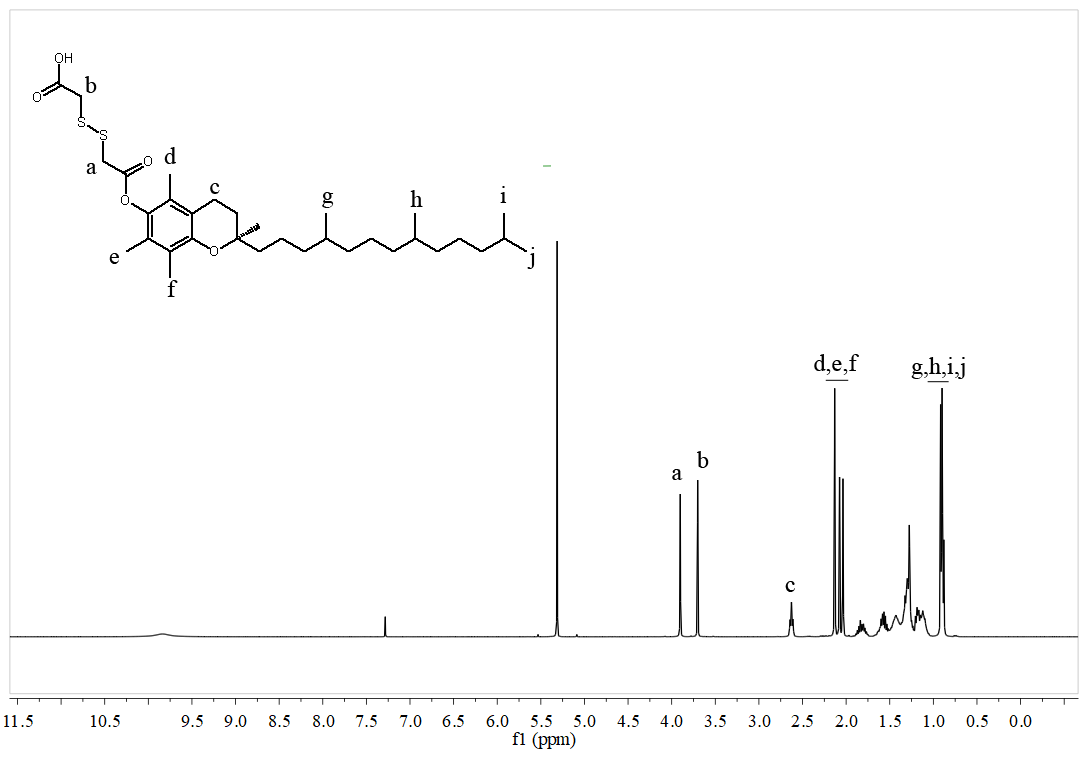


Figure S1 ^1^H-NMR of VE-S-S-COOH (CDCl_3_, ppm)


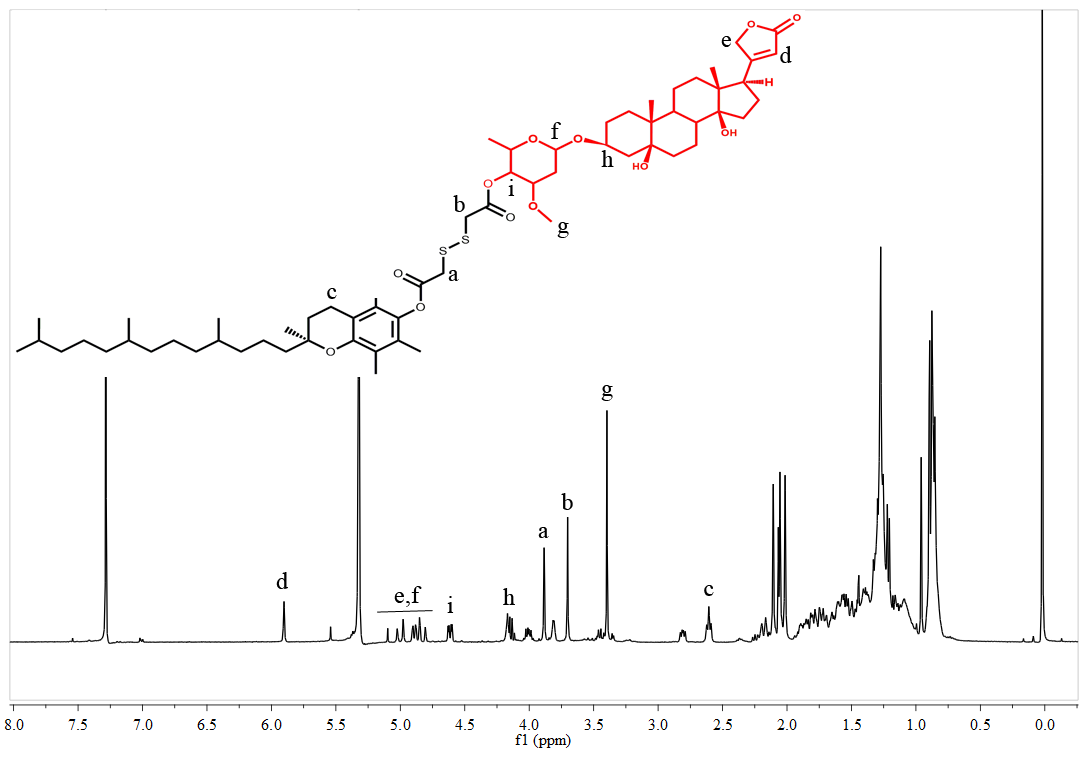


Figure S2 ^1^H-NMR of PPM-S-S-VE. (CDCl_3_, ppm)


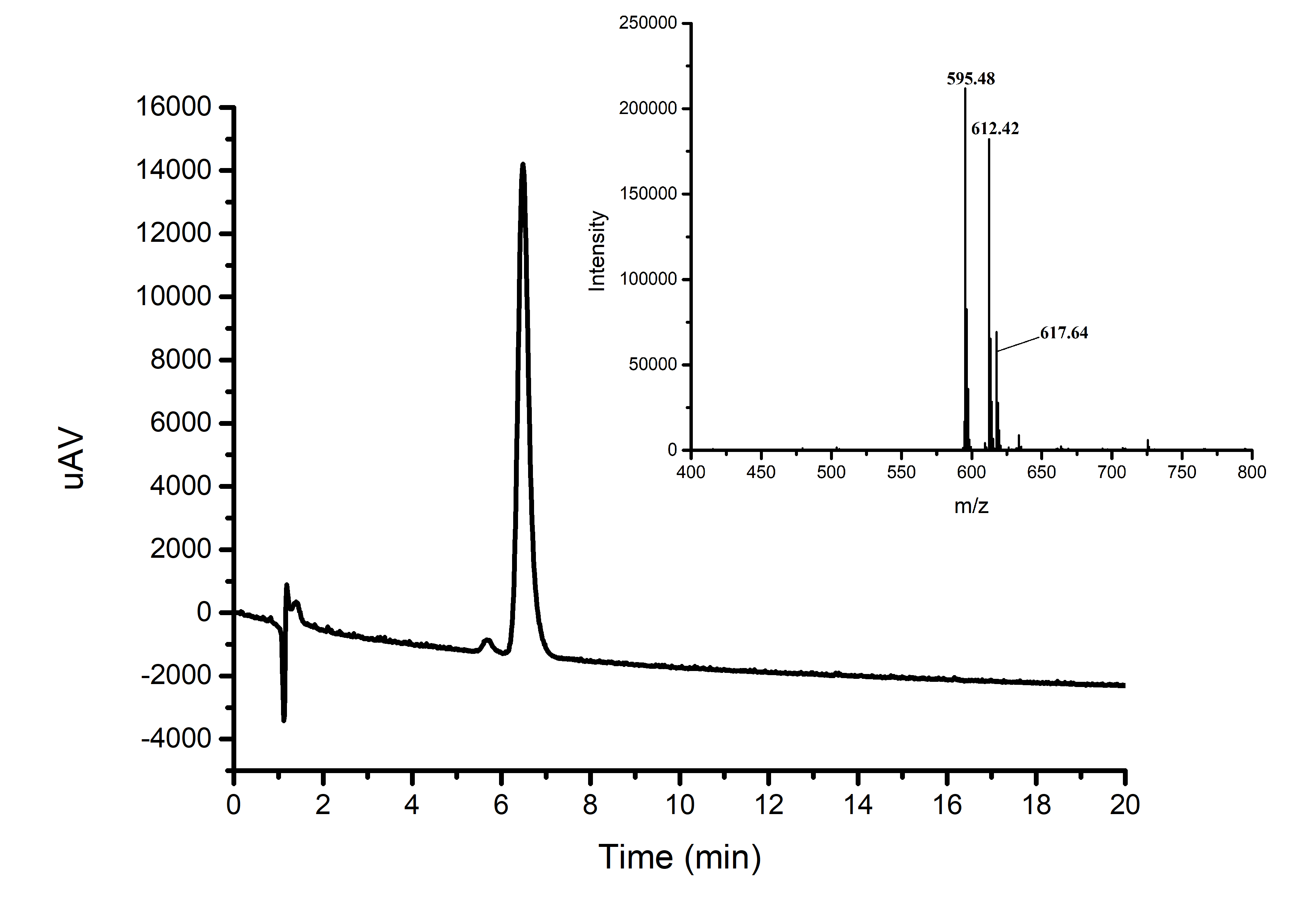


Figure S3 ESI-MS and HPLC chromatographic of VE-S-S-COOH


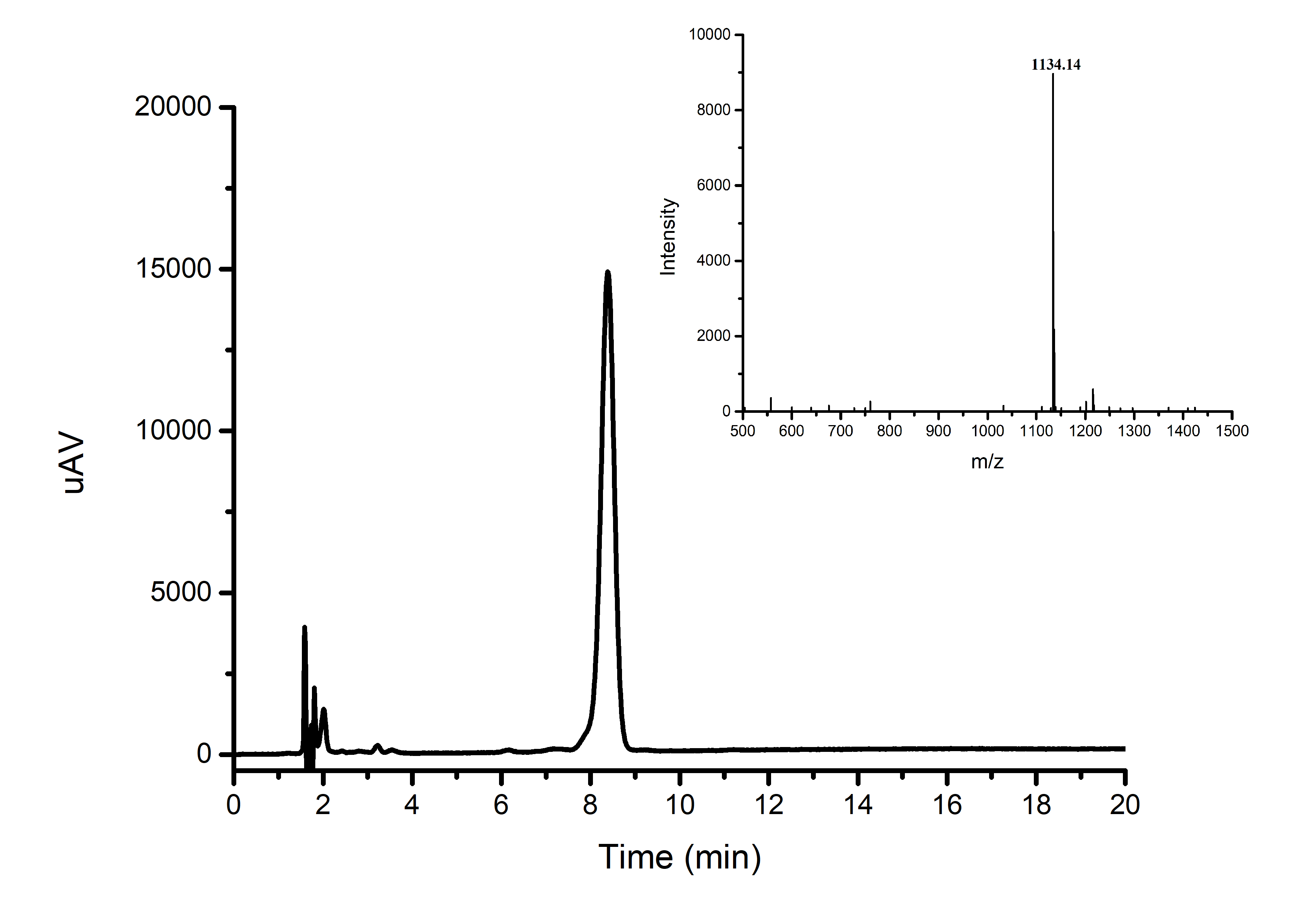


Figure S4 ESI-MS and HPLC chromatographic of PPM-S-S-VE


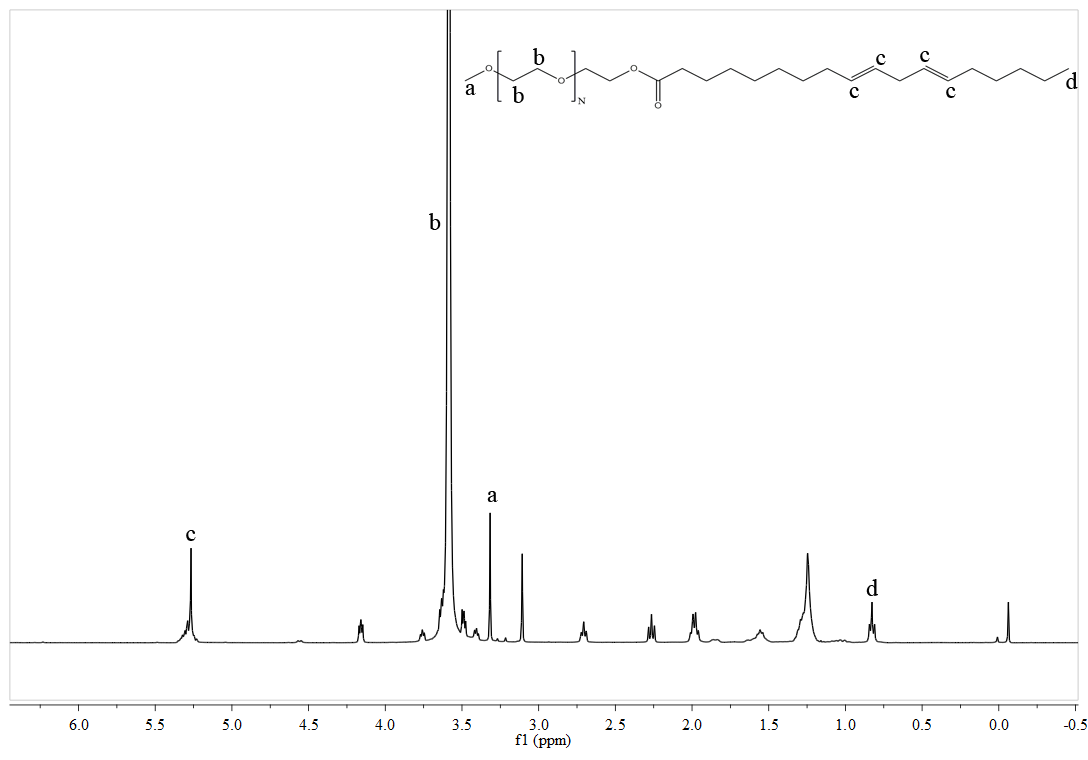


Figure S5 ^1^H-NMR of mPEG_2000_-LA (CDCl_3_, ppm)

Figure S6 Q-TOF-MS characterization of mPEG2000

Figure S7 Q-TOF-MS characterization of mPEG2000-LA


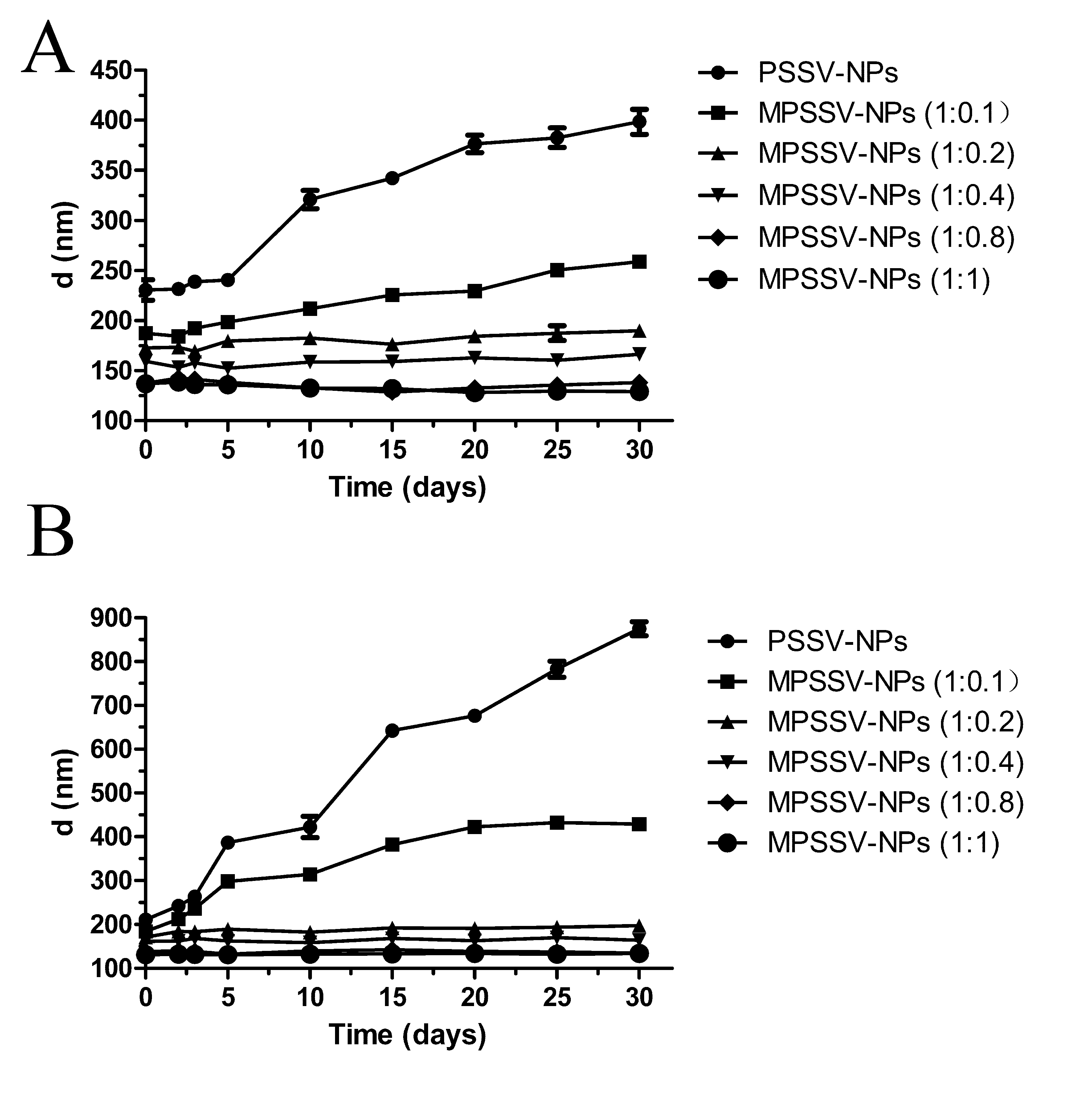


Figure S8. Stability in water (A) and PBS (B) of PSSV-NPs and MPSSV-NPs with different ratios (1 : 0.1, 1 : 0.2, 1 : 0.4, 1 : 0.8, 1: 1). The values are represented as mean ± standard deviation of three determinations


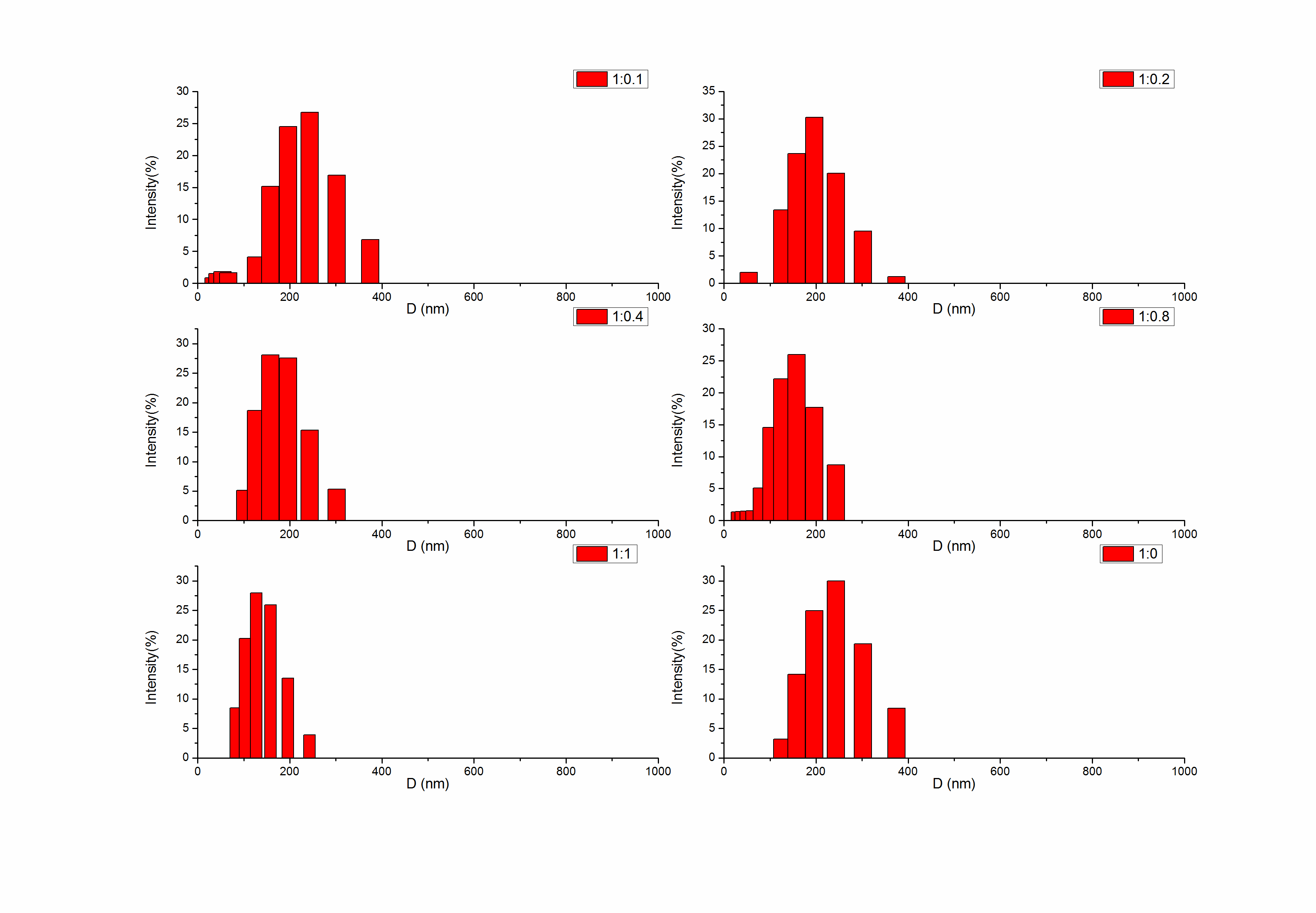


Figure S9 DLS profiles of the nanoassemblies made of mixtures of PPM-S-S-VE or mPEG-LA


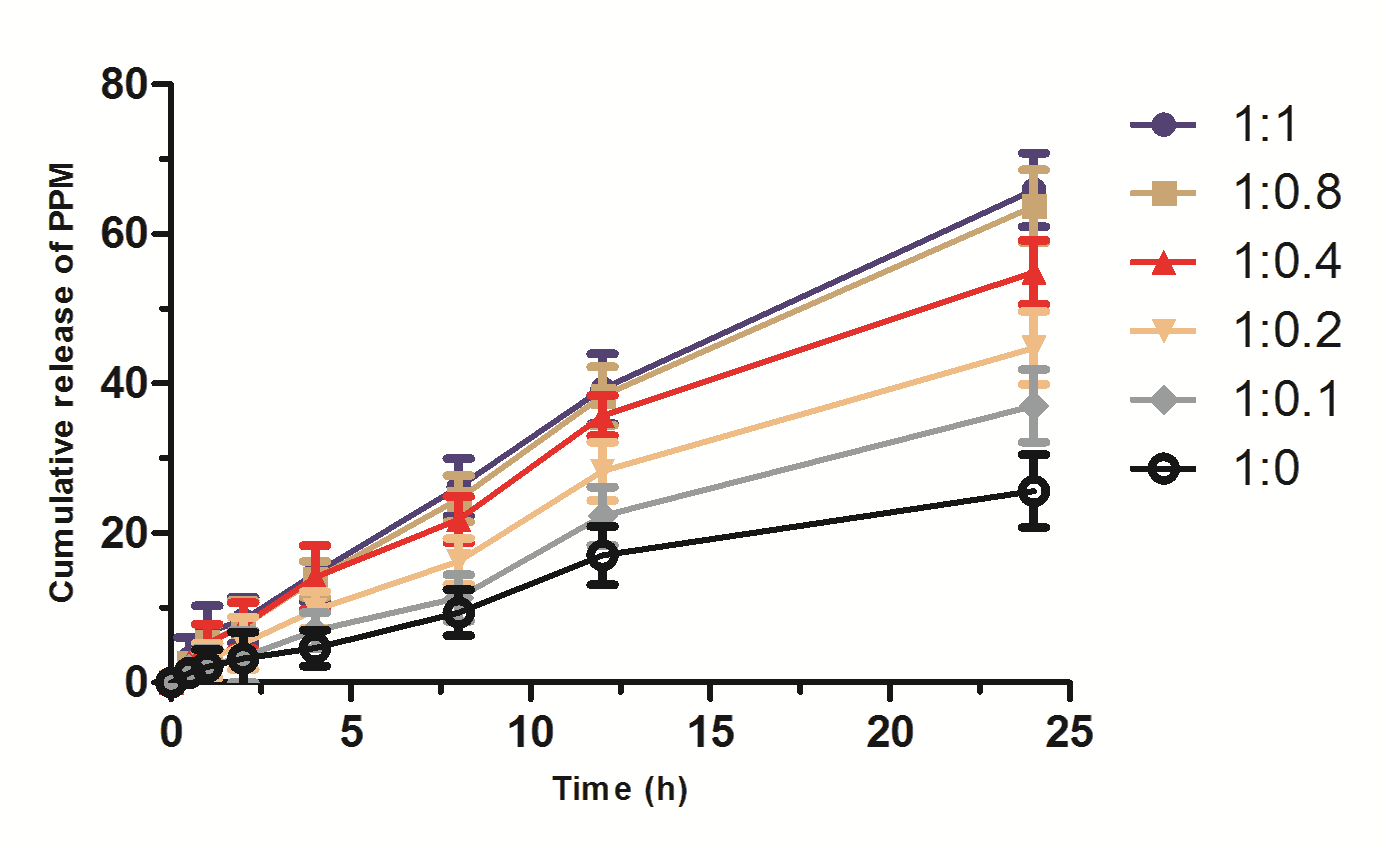


Figure S10. In *vitro* release study of the nanoassemblies made of mixtures of PPM-S-S-VE or mPEG-LA (mean values ± standard deviation, n=3) in 1mM GSH medium


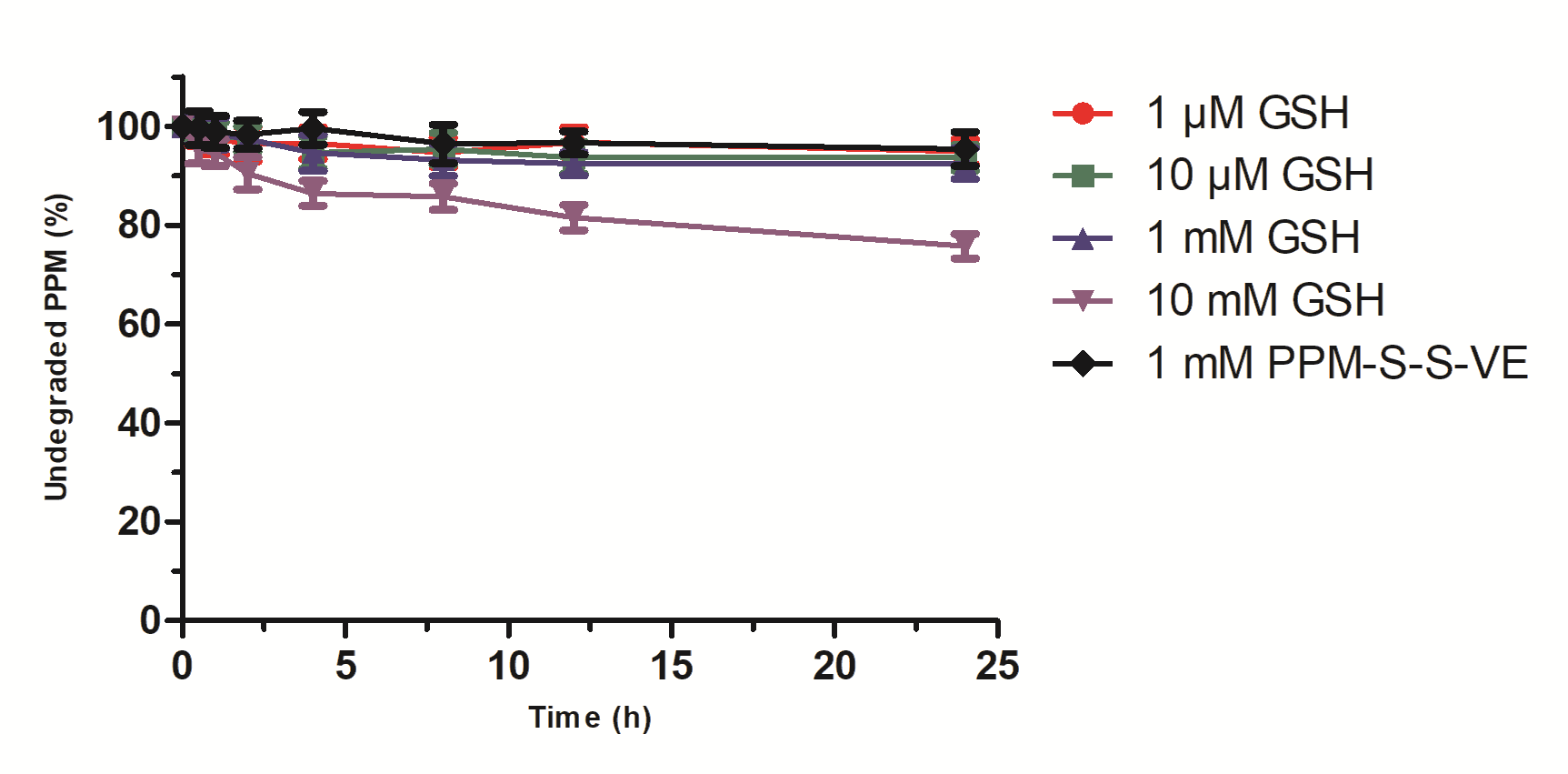


Figure S11 Stability of PPM were studied at 37 °C under four different conditions, i.e, 10 mM, 1 mM, 10 μM, and 1 μM GSH, respectively (n= 3)


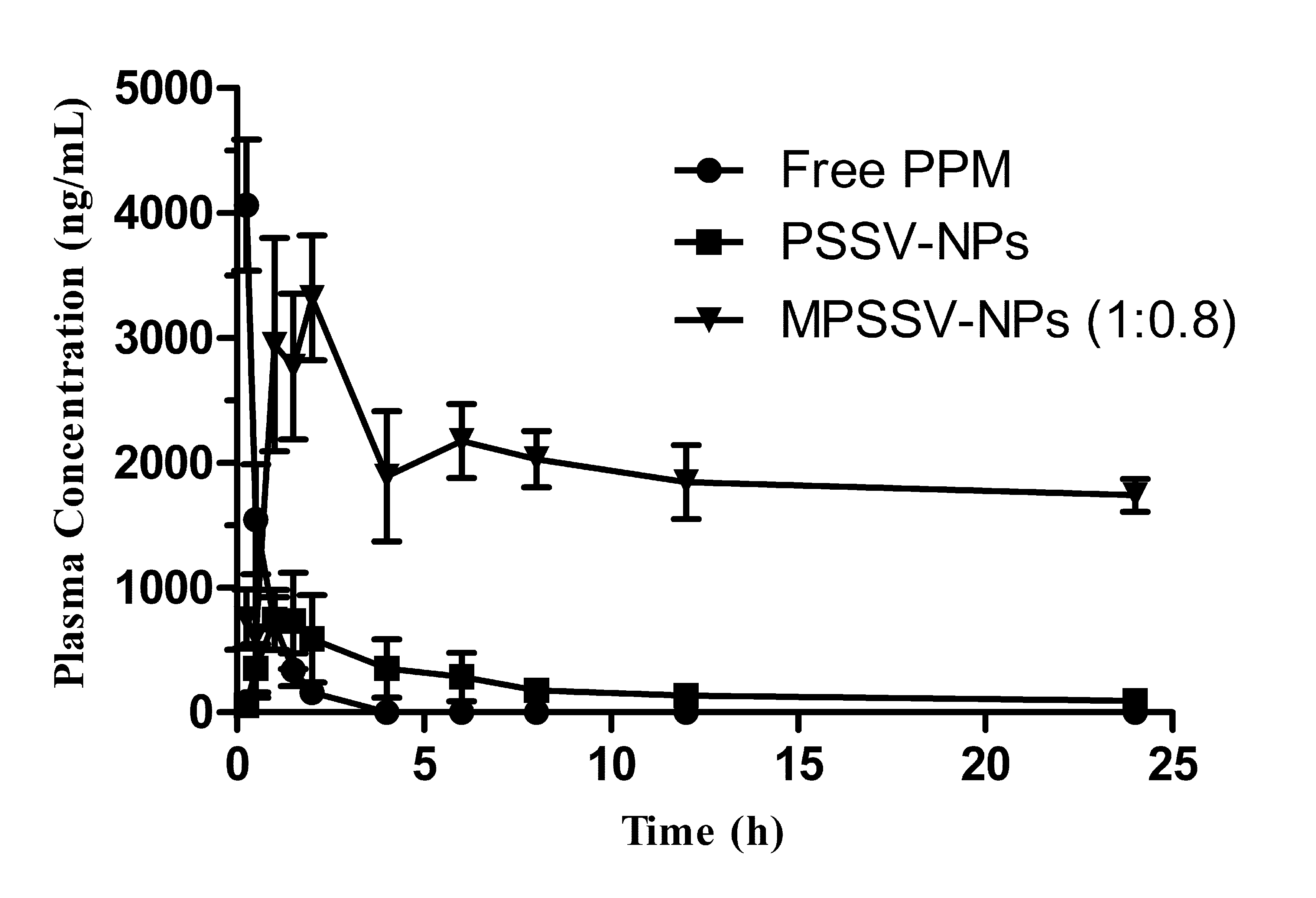


Figure S12. *In vivo* pharmacokinetics of PSSV-NPs and MPSSV-NPs after intravenous administration in rats. (Error bars are mean ± SD, n = 5)

Table S1. Physicochemical characterization of the nanoassemblies made of mixtures of PPM-S-S-VE or mPEG-LA (mean values ± standard deviation, n=3): measurement of mean diameter (d), Zeta potential (z) and polydispersity index (PDI).

| Type of nanoassemblies | Ratio | Z[mV] | D[nm] | PDI |
| --- | --- | --- | --- | --- |
| VE-S-S-PPM | 1︰0 | -28.42 ± 1.23 | 230.54 ± 10.23 | 0.085 ± 0.008 |
| VE-S-S-PPM︰LA-PEG | 1︰0.1 | -26.74 ± 1.03 | 187.34 ± 2.53 | 0.088 ± 0.016 |
|  | 1︰0.2 | -25.31 ± 1.24 | 172.79 ± 2.42 | 0.120 ± 0.012 |
|  | 1︰0.4 | -24.27 ± 1.34 | 159.33 ± 2.70 | 0.150 ± 0.013 |
|  | 1︰0.8 | -23.16 ± 1.12 | 137.06 ± 1.74 | 0.189 ± 0.010 |
|  | 1:1 | -23.09 ± 1.20 | 131.32 ± 2.49 | 0.190 ± 0.11 |

Table S2 *In vitro* cytotoxicity of PSSV-NPs and MPSSV-NPs with various concentrations on HepG2 and MCF-7 cells after 72 h of incubation, IC_50_ μM)

| NPs | MCF-7 | | HepG2 | |
| --- | --- | --- | --- | --- |
|  | IC_50_ | 95%CI | IC_50_ | 95%CI |
| PPM | 0.036 | 0.025-0.051 | 0.138 | 0.089-0.213 |
| PSSV-NPs | 4.517 | 2.962-6.889 | 0.068 | 0.243-0.467 |
| MPSSV-NPs | 0.090 | 0.065-0.124 | 3.257 | 3.241-7.904 |

Table S3 Pharmacokinetic parameters of PSSV-NPs and MPSSV-NPs after intravenous administration in rats (mean ± SD)

| Parameters | Free drug | PSSV-NPs | MPSSV-NPs |
| --- | --- | --- | --- |
| C_max_ (ng∙mL^-1^) | 4060 ±524 | 743.5 ± 239 | 3318.9 ± 498^1^ |
| T_max_ (h) | 0.25 | 1 | 2 |
| t_1/2_ (h) | 0.327 ± 0.03 | 3.339 ± 0.37^2^ | 30.04 ± 1.27^2^ |
| MRT (h) | 0.635 ± 0.09 | 9.487 ± 0.84^2^ | 45.87 ± 1.62^2^ |
| AUC_0-4h_ (h∙ng∙mL^-1^) | 2159 ± 184.2 | 5057 ± 286^2^ | 46817 ± 3184^2^ |

^1^p < 0.05, compared with free PPM

^2^p< 0.01, compared with free PPM
